# N-MYC regulation of DNA damage response in neuroendocrine prostate cancer: mechanistic insight and novel combination therapy approaches

**DOI:** 10.18632/oncoscience.462

**Published:** 2018-08-22

**Authors:** Timothy C. Thompson, Bo Liu, Likun Li

**Affiliations:** Department of Genitourinary Medical Oncology, The University of Texas MD Anderson Cancer Center, Houston, TX 77030-4009, USA

**Keywords:** DNA damage response, neuroendocrine prostate cancer, N-MYC, PARP inhibitors, Combination therapy

Accumulation of DNA damage leads to genomic instability and can drive cancer progression. Germline and somatic mutations in the BRCA1 or BRCA2 genes can contribute to this genomic instability on the basis of their role in DNA repair. Both BRCA1 and BRCA2 are critical for repair of DNA double-strand breaks by homologous recombination (HR), a conservative form of DNA repair [1]. HR is a critical component of the DNA damage response (DDR) signaling cascade, which detects and propagates DNA damage signals to elicit cellular responses that include cell cycle arrest, DNA repair, and apoptosis. The activities of the DDR signaling cascade are required for DNA-damaged cells to complete the cell cycle, survive and proliferate. Metastatic castration-resistant prostate cancer (CRPC) is associated with increased frequency of germline and somatic DDR gene mutations, including BRCA2, suggesting that DDR targeting therapy provides therapeutic options through synthetic lethal strategies such as poly(ADP-ribose) polymerase (PARP) inhibition [1, 2]. However, CRPC can transition to a more virulent, and poorly differentiated form of the CRPC, i.e., CRPC-Neuro or neuroendocrine prostate cancer (NEPC). Similar to other poorly differentiated neuroendocrine tumors, NEPC can exhibit small cell morphology and neuroendocrine differentiation, and responds poorly to existing therapies [3].

Previous studies have shown that DNA repair deficiency represented by a low recombination proficiency score was associated with increased expression of a broad array of homologous recombination (HR)-related genes, suggesting compensation of DNA repair defects through overexpression of functional DDR genes [4]. We demonstrated increased HR gene expressions in CRPC compared to hormone naïve prostate cancer, supporting a functional role for increased DDR gene expression in CRPC [5]. Given that androgen receptor inhibitor-induced HR gene suppression, or genetic HR gene knockdown can potentiate the effects of PARP inhibition in prostate cancer models with variable androgen dependence [5], it is reasonable to assume that increased DDR gene expression plays a role in maintaining DNA repair and cell survival in CRPC, and may represent critical tumor dependency pathways. In this context increased DDR gene expression may also reflect DDR deficiencies in specific DNA repair pathway(s), and signal an altered response to and DNA damaging chemotherapy including platinum-based agents [4]. Functional analysis of specific DDR pathways—in addition to DDR gene mutations— may reveal useful biomarkers and therapy targets for CRPC. Greater mechanistic understanding of these DDR pathways and their regulation may provide a way forward in the development of new treatment options for CRPC and NEPC.

MYCN and its binding partner AURKA have been shown to play an important role in NEPC, and MYCN is overexpressed in a subset of CRPC adenocarcinomas (CRPC-Adeno). In a recent publication we identified a novel oncogene-driven DDR signaling pathway that can compensate, and potentially rescue prostate cancer cells from debilitating DNA damage repair deficiencies. We identified a MYCN-PARP-DDR pathway, and showed that it is driven by N-MYC transcriptional regulation of PARP1, PARP2, and DDR genes [6]. Importantly, the MYCN-regulated DDR genes identified in this study include HR genes (BRCA1, RMI2), and a replication stress-related gene (TOPBP1). Genetic knockdown of MYCN led to reduced expression of these genes and increased DNA damage in CRPC-Adeno and CRPC-Neuro prostate cancer cells. Identification of this pathway adds a new dimension to the molecular phenotype for NEPC and points to a novel panel of DDR-related candidate prognostic biomarkers for the transition from CRPC-Adeno to CRPC-Neuro. In addition, identification of the MYCN-PARP-DDR pathway establishes a predictive biomarker—informed actionable target for novel combination therapy strategies. We demonstrated the feasibility of suppressing MYCN-PARP-DDR signaling by using an AURKA inhibitor (PHA739358) and a PARP inhibitor (olaparib) in preclinical NEPC models.

**Figure 1:**
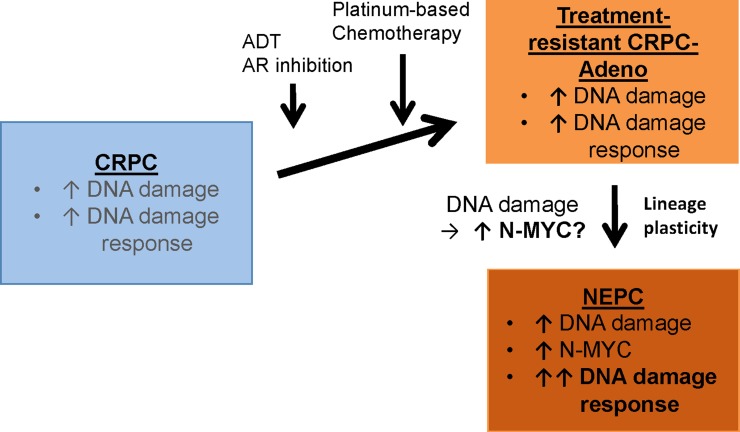
Association of increased N-MYC-mediated DNA damage response with neuroendocrine prostate cancer Androgen deprivation therapy (ADT), AR inhibition therapy, and platinum-based chemotherapy leads to treatment-resistant, castration-resistant prostate cancer (CRPC). A subset of these cancers progress to neuroendocrine prostate cancer (NEPC), a highly metastatic disease that can exhibit small cell morphology and neuroendocrine differentiation. Zhang et al. recently demonstrated increased DNA damage response (DDR) gene expression in NEPC compared to CRPC with adenocarcinoma morphology, and further identified the MYCN-PARP-DDR pathway driven by N-MYC transcriptional regulation of PARP1, PARP2, and DDR genes [6]. This report links NEPC with increased DDR expression and establishes a novel N-MYC-driven molecular pathway for biomarker development and targeted therapy approaches. The MYCN-PARP-DDR signaling pathway identified in NEPC may be required to contain the deleterious effects of DNA damage that might occur prior to and during the transition from CRPC-Adeno to CRPC-Neuro.

In addition to identifying novel opportunities for therapy, identification of a direct regulatory connection between N-MYC and DDR in NEPC provokes questions related to the potentiality of MYCN and the MYCN-PARP-DDR pathway in CRPC cell survival and lineage plasticity. We showed that MYCN knockdown suppressed the MYCN-PARP-DDR pathway and led to increased DNA damage in NEPC [6]. Interestingly, recent publications that show N-MYC regulates expression of three components of the MRE11/RAD50/NBS1 (MRN) complex demonstrate the role of N-MYC in the molecular control of replication stress, proliferation, and cell survival in granule cell progenitor expansion and differentiation during cerebellar development [7]. It's conceivable that the MYCN-MRN complex axis could play a comparable regulatory role prior to and during the transition from CRPC-Adeno to CRPC-Neuro. In addition to MYCN overexpression, genomic alterations in the *TP53* and *RB1* tumor suppressor genes, are associated with the evolution of NEPC, and functional inactivation of these genes has been shown to promote lineage plasticity in genetically engineered mouse models (reviewed in [3]). Within the context of loss of *TP53* and *RB1* function, it is tempting to speculate that the MYCN-PARP-DDR signaling pathway is necessary to contain the deleterious effects of DNA damage that occur as a result of loss of cell cycle checkpoint control prior to and during the transition from treatment-resistant CRPC-Adeno to CRPC-Neuro (Figure 1). Additional studies will be required to address these and other questions related to the complex role of MYCN in NEPC.
